# Neuroendocrine carcinoma of the gallbladder: A case report and literature review

**DOI:** 10.1097/MD.0000000000039147

**Published:** 2024-08-02

**Authors:** Xiaofei Yao, Kangze Wu, Baochun Lu, Feizhuan Lin

**Affiliations:** a Pathology Department, Shaoxing People’s Hospital, Shaoxing, People’s Republic of China; b Department of Hepatobiliary Surgery, Shaoxing People’s Hospital, Shaoxing, People’s Republic of China.

**Keywords:** gallbladder, immunohistochemistry, liver metastasis

## Abstract

**Rationale:**

Neuroendocrine neoplasms (NENs) originating from neuroendocrine cells occur in the thyroid, respiratory, and digestive systems, with Gallbladder Neuroendocrine Carcinoma (GB-NEC) accounting for only 0.5% of all NENs and 2.1% of gallbladder cancers. Due to its rarity, little is known about GB-NEC’s clinical presentation and treatment.

**Patient concerns:**

We report a case of a 52-year-old male presenting with acute upper right abdominal pain, leading to further investigation.

**Diagnoses:**

Initial diagnostic workup, including abdominal ultrasound and contrast-enhanced CT, suggested gallbladder malignancy. Post-surgical pathology confirmed GB-NEC, with immunohistochemistry supporting the diagnosis.

**Interventions:**

The patient underwent radical cholecystectomy, followed by etoposide plus cisplatin chemotherapy. After disease progression indicated by CT, the patient received additional cycles of chemotherapy with cisplatin and irinotecan, plus targeted therapy with anlotinib and immunotherapy with paimiplimab.

**Outcomes:**

The patient showed a partial response to initial treatment. Subsequent liver biopsy confirmed NEC, consistent with small cell carcinoma. With continued treatment, the patient maintains a good survival status.

**Lessons:**

GB-NEC is associated with poor prognosis, emphasizing the importance of early detection and multimodal treatment strategies. Our case underlines the potential benefit of a comprehensive treatment plan, including aggressive surgery and chemotherapy, with further research needed to standardize treatment for this rare condition.

## 1. Introduction

Tumors that arise from neuroendocrine cells and are seen in the thyroid, respiratory, and digestive systems are known as neuroendocrine neoplasms (NENs).^[1]^ Merely 0.5% of all NENs and 2.1% of all gallbladder cancers were caused by GB-NEC (Gallbladder Neuroendocrine Carcinoma).^[2]^ Many clinical questions regarding GB-NEC remain unanswered in the literature due to its low incidence rate. Herein, we report a rare case of small-cell neuroendocrine carcinoma of the gallbladder with concurrent liver metastasis and give a brief review of the literature. Small-cell neuroendocrine carcinoma of the gallbladder is extremely rare, but it is even rarer for this case to present with liver metastasis after a year and a half. This case highlights that a comprehensive treatment approach centered on surgery can significantly improve the prognosis of patients with GB-NEC.

## 2. Case report

**Figure 1.**
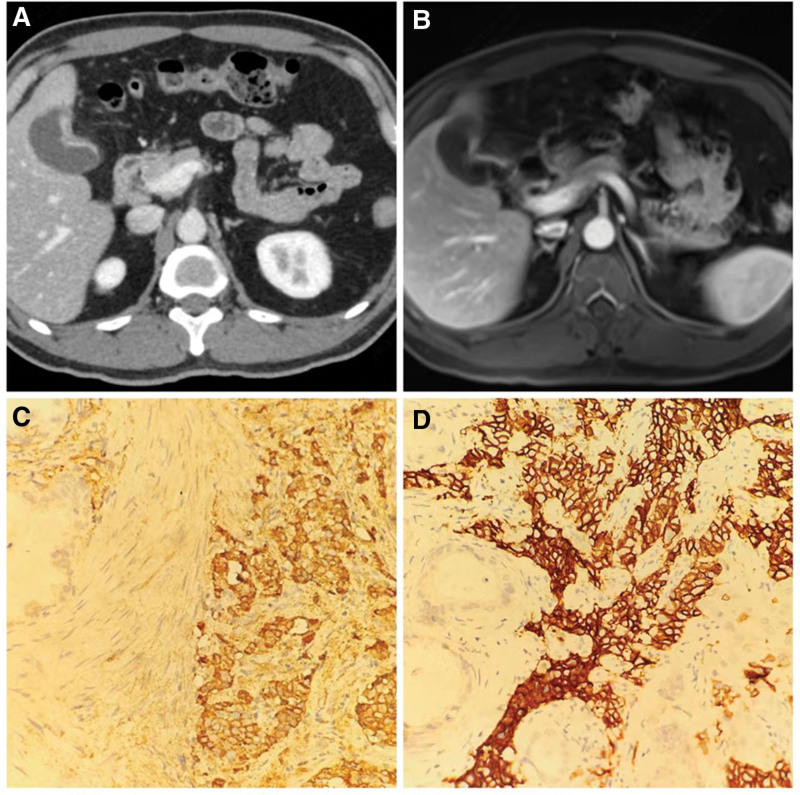
(A) Contrast-enhanced computed tomography scan showing localized irregular thickening of the gallbladder wall. (B) MRI indicating local thickening of the gallbladder wall, raising suspicion for malignancy. (C) IHC staining showing CgA (+). (D) IHC staining showing CD56 (+). CgA = chromogranin A, IHC = immunohistochemical, MRI = magnetic resonance imaging.

A 52-year-old male patient presented to the hospital with a complaint of upper right abdominal pain lasting for two days without any other discomfort. Physical examination revealed tenderness in the upper right quadrant of the abdomen but no rebound tenderness and no other positive signs. Laboratory tests yielded no positive findings. The patient’s carcinoembryonic antigen (CEA) and carbohydrate antigen 19-9 (CA199) levels were within normal ranges. An abdominal ultrasound was then performed, which indicated a rough gallbladder wall, an echo-poor mass within the gallbladder, and possible sediment. Further abdominal contrast-enhanced computed tomography (CT) suggested localized irregular thickening of the gallbladder wall (Fig. 1A). Magnetic resonance imaging (MRI) also indicated local thickening of the gallbladder wall, raising suspicion for malignancy (Fig. 1B). After discussion, it was decided to perform a radical cholecystectomy for suspected gallbladder cancer. Postoperative pathology reported: malignant tumor consistent with neuroendocrine carcinoma (NEC) with moderate differentiation adenocarcinoma (5%), infiltrating the outer serous fibro-fatty tissue, and nerve involvement visible in the lesion area. Immunohistochemical (IHC) staining showed: Ki-67 (+70%), cytokeratin 7 (CK7) (+), epithelial membrane antigen (EMA) (+), CKpan (+), leukocyte common antigen (LCA) (−), synaptophysin (Syn) (weakly +), chromogranin A (CgA) (+), and CD56 (+) (Fig. 1C and D). Lymph nodes at the base of the gallbladder were considered to be involved in neuroendocrine carcinoma.

**Figure 2.**
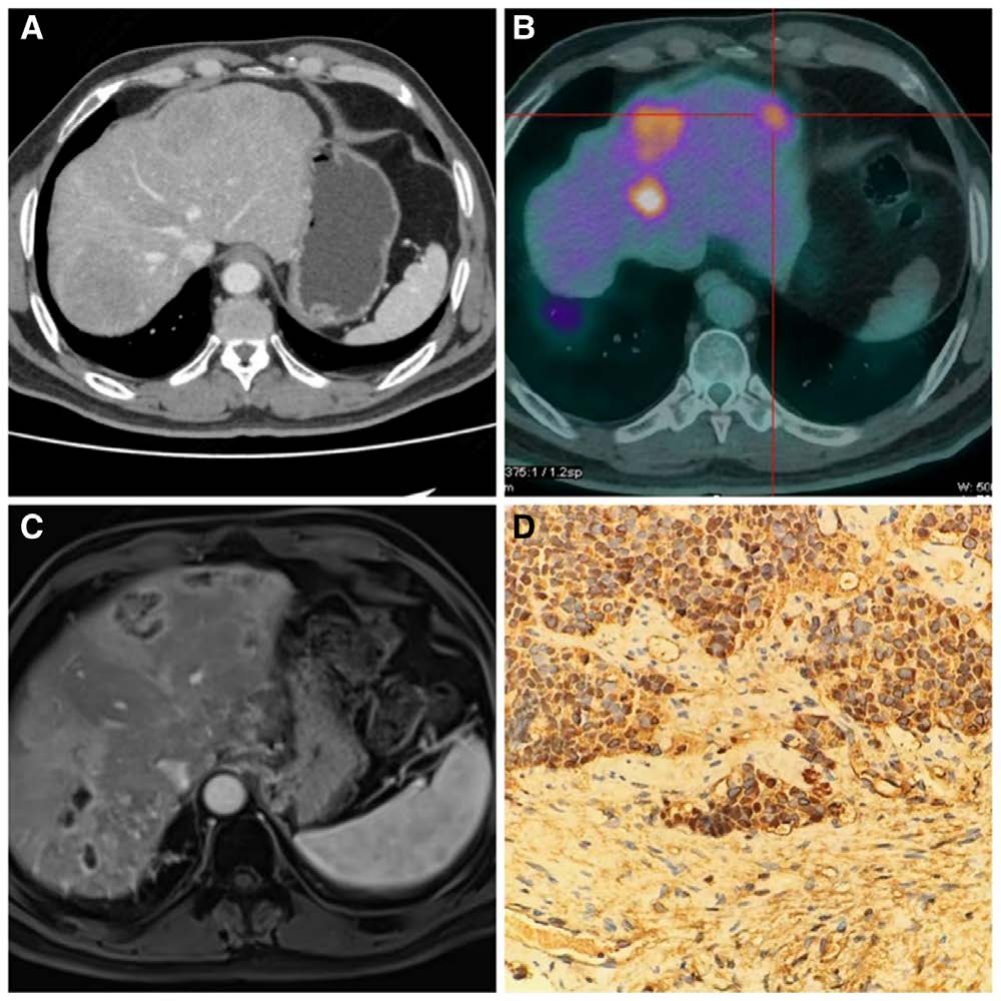
(A) CT scan 16 months post-surgery revealing multiple masses in the liver and a filling defect in the left branch of the portal vein suggesting a tumor thrombus. (B) PET-CT scan showing multiple masses and nodules in the liver with increased FDG metabolism, indicative of multiple hepatic metastatic tumors. (C) Magnetic resonance scan showing an increase in size of some metastatic tumors in the liver and persistent tumor thrombus in the portal vein. (D) IHC results from a liver biopsy indicating neuroendocrine carcinoma with the following staining: CgA (+). CgA = chromogranin A, CT = computed tomography, FDG = fluorodeoxyglucose, IHC = immunohistochemical, PET-CT = positron emission tomography-computed tomography.

Postoperatively, the patient was treated with etoposide plus cisplatin chemotherapy, and multiple follow-up CT scans did not show any evident metastatic lesions. Approximately 16 months after the surgery, the patient returned for a CT scan, which revealed multiple masses in the liver and a filling defect in the left branch of the portal vein, suggesting a tumor thrombus (Fig. 2A). Laboratory tests also indicated a CA199 level of 90.4 U/mL. After discussion, a positron emission tomography-computed tomography (PET-CT) was performed, the results of which showed multiple masses and nodules in the liver with increased fluorodeoxyglucose (FDG) metabolism (Fig. 2B), further pointing to the diagnosis of multiple hepatic metastatic tumors. Subsequently, the patient underwent six cycles of chemotherapy with cisplatin and irinotecan, supplemented with anlotinib and paimiplimab.

During treatment, an assessment of therapeutic effect showed a partial response (PR). However, an MR scan conducted on November 23 revealed that some of the metastatic tumors in the liver had increased in size compared to before, and the tumor thrombus in the portal vein was still present, implying further progression of the tumor (Fig. 2C). To gain further pathological evidence, a liver puncture biopsy was performed on the patient, and the pathology indicated neuroendocrine carcinoma (consistent with small cell carcinoma). The IHC results were as follows: alpha-fetoprotein (AFP) (−), hepatocyte (−), Ki-67 (+, 20%), CK19 (+), CK20 (−), CK7 (−), CgA (+), Syn (−), and CD56 (+) (Fig. 2D). After thorough discussion with the patient, he started treatment with anlotinib hydrochloride injection on November 30, undergoing 4 treatments. Currently, the patient is still undergoing regular follow-ups and has a good survival status.

## 3. Discussion

Tumors derived from neuroendocrine cells or exhibiting neuroendocrine differentiation are known as NENs, and their yearly incidence ranges from 2 to 7 cases per 100,000 individuals.^[2,3]^ Rare and aggressive neuroendocrine cancer originating from the gallbladder is known as GB-NEC.^[4]^ According to statistics from the surveillance epidemiology and end result (SEER) study, 2.1% of all gallbladder tumors and around 0.5% of all NENs are GB-NECs, which is an incredibly rare condition.^[2]^ In 2019, the WHO proposed a new classification system that splits GB- NENs into three categories: neuroendocrine carcinomas (NECs, large cell or small cell type), NET (grade 1, 2, or 3 NETs), and mixed neuroendocrine-non-neuroendocrine neoplasms (MiNENs).^[5]^

Since neuroendocrine cells are absent from the normal gallbladder mucosa, the mechanisms behind the occurrence of GB-NET remain unclear.^[6]^ The following theories have been put forth by earlier research: undifferentiated gallbladder stem cells give rise to neuroendocrine cells^[7]^; pathological intestinal metaplasia, resulting from prolonged chronic inflammation of the gallbladder mucosa, produces neuroendocrine cells at the lesion site and progresses to neuroendocrine carcinoma^[8]^; and the function of gallbladder adenocarcinoma may occasionally change to a neuroendocrine one.^[4,9,10]^

According to the literature we have reviewed, GB-NEC appears to be more prevalent in female patients (Table 1). Reports suggest a male-to-female ratio of approximately 1:2, with an average age of diagnosis of about 68 years.^[19]^ It is possible that these findings may still be subject to bias due to the limited number of cases. A median of seven months is the overall survival (OS).^[19]^ Based on how the tumor produces peptides, GB neuroendocrine carcinomas can be classified as secretory or non-secretory.^[20]^ Nonfunctioning tumors commonly present signs of malignancy, such as cachexia, jaundice, fatigue, and right upper quadrant pain. Functional tumors, due to their ability to produce hormones like serotonin, CgA, adrenocorticotropic hormone (ACTH), and gastrin, may exhibit hormone-related side effects, such as flushing, diarrhea, and coughing.^[15,21,22]^ Most cases of gallbladder cancer do not exhibit typical clinical signs or symptoms in the early stages, and as such, they are often found at an advanced stage. Consequently, fewer than 20% of patients are eligible for a complete surgical resection. In this case, the patient presented to the hospital due to upper abdominal pain, which allowed for a for a timely preoperative examination, malignancy consideration, and subsequent surgical resection.

**Table 1:** Summary of neuroendocrine carcinoma of the gallbladder from the literature.

Study	Age	Gender	Symptom	Intervention	Ki-67	Adjuvant treatment	Outcomes	IHC
Park et al^[11]^	49	F	No	Radical cholecystectomy	30	Yes	Unknown	Not available
	66	M	No	Radical cholecystectomy	25	No	Unknown	Not available
	74	F	No	Radical cholecystectomy	30	No	Unknown	Not available
Majumder and Dhakane^[12]^	58	F	Pain	Laparoscopic cholecystectomy	Unknown	Unknown	Unknown	Pan-cytokeratin (+), CD56 (+), S100 (+), chromogranin (+), Syn (+)
Arai et al^[13]^	76	F	Unknown	Radical cholecystectomy	Unknown	Unknown	5 mo	Not available
Liu et al^[14]^	69	F	Pain	Laparoscopic cholecystectomy with gallbladder bed cautery	80	No	29 mo	CgA (+), Syn (+)
Adachi et al^[10]^	79	F	Pain	Radical cholecystectomy	Unknown	Unknown	13 wk	Chromogranin (+), Syn (−)
Chen et al^[15]^	65	F	Unknown	Radical cholecystectomy	Unknown	No	170 d	CgA (+), NSE (+), CK (+)
	49	F	Unknown	Radical cholecystectomy	Unknown	Yes	700 d	CgA (+), NSE (+), CK (+)
Yazawa et al^[16]^	60	F	Unknown	Radical cholecystectomy	Unknown	Unknown	8 yr	Not available
Bhatia et al^[17]^	36	F	Jaundice	Radical cholecystectomy	80	Unknown	Unknown	Chromogranin (+), NSE (+), CK (+)
Wang et al^[18]^	56	F	No	Radical cholecystectomy	50	Yes	15 mo	Syn (+), CgA (+), CD56 (+), CK7 (−), CK19 (−),CK(+)

Pathological diagnosis is the gold standard since neuroendocrine tumors of the gallbladder do not exhibit characteristic radiographic characteristics. Pathological and IHC analyses using markers as Syn, neuron-specific enolase (NSE), and CHG-A are necessary for the diagnosis of GB-NEN.^[7]^ The positive rates for Syn, EMA, and CD56 are relatively low.^[15]^ Our case demonstrated positivity for CK, Syn, CgA, and CD56, thereby confirming the diagnosis of a gallbladder neuroendocrine tumor.

In the treatment of GB-NEC, surgical resection remains the preferred and primary treatment method, including simple cholecystectomy, palliative cholecystectomy, and radical cholecystectomy.^[11]^ There is disagreement over the best ways to treat GB-NEC because of its incredibly low prevalence. According to some academics, patients who have received adjuvant chemotherapy and those who have not shown no appreciable differences.^[23]^ The European Neuroendocrine Tumor Society (ENETS) 2023 guidelines for digestive neuroendocrine carcinoma state that the best evidence supports irinotecan plus fluoropyrimidines as the second-line treatment, while platinum in combination with etoposide is advised as the first-line treatment for patients with metastatic GB-NEC.^[24,25]^ According to our experience, aggressive surgery, chemotherapy administered after the procedure, and prompt modification of treatment plans can all assist to halt the disease’s progression.

The prognosis of GB-NEC is poorer than that of gallbladder adenocarcinoma. Some scholars have used propensity score matching to compare the overall OS of patients with GB-NEC and gallbladder adenocarcinoma. The results suggest that the OS of GB-NEC patients is significantly lower than that of gallbladder adenocarcinoma patients, with median OS (mOS) of 15.02 and 20.11 months, respectively.^[26]^ Patients with gallbladder neuroendocrine tumors have poor prognoses, with a mOS of 20.20 (17.99*–*22.41) months for those receiving multimodal therapy, compared to 4.00 (2.91–5.10) months for those not receiving multimodal therapy.^[27]^ Surgery by itself was linked to improved short-term (<2 years) result survival, whereas surgery in combination with radiation and adjuvant chemotherapy was related with better long-term (5 years) outcomes.^[28]^

## 4. Conclusions

The prognosis for GB-NEC is poor, making early detection crucial for patient outcomes. Following diagnosis, multimodal treatments, including surgery, can improve survival rates for these patients. Essential pathological examinations are key for diagnosing neuroendocrine tumors. Due to the rarity of GB-NEC cases, there is currently a lack of consensus on specific treatment strategies, such as chemotherapy and targeted immunotherapy. Therefore, more extensive prospective research is needed in the future.

## Author contributions

**Conceptualization:** Xiaofei Yao, Feizhuan Lin.

**Data curation:** Xiaofei Yao, Kangze Wu, Baochun Lu.

**Writing – original draft:** Xiaofei Yao, Feizhuan Lin.

**Writing – review & editing:** Feizhuan Lin.
